# Governments should show climate and health ambition in their nationally determined contributions

**DOI:** 10.1136/bmj.r2217

**Published:** 2025-11-07

**Authors:** Jess Beagley, Fabio Cresto Aleina, Andy Haines

**Affiliations:** 1 Global Climate and Health Alliance; 2 Centre on Climate Change and Planetary Health, London School of Hygiene and Tropical Medicine

The world is getting hotter. Current emission reduction efforts set the world on course to reach about 3.1°C above pre-industrial temperatures by 2100. Even if countries take further action and fully deliver on existing national climate targets for 2030, this would still lead to an increase of 2.3-2.5°C.^1^ Temperature rises of this scale would cause catastrophic climate and health harms. Already, many countries’ healthcare systems are under pressure and struggling to deliver universal health coverage.^2^

Without immediate and transformative global action to reduce greenhouse gas emissions, worsening health hazards will rapidly exceed the limits of adaptation in many settings. The world’s highest greenhouse gas emitting countries and biggest economies need to make the largest reductions to their emissions. G20 member states (excluding the African Union) were responsible for 77% of greenhouse gas emissions in 2024, whereas least developed countries accounted for only about 3%.^1^ If countries were to take ambitious climate mitigation and adaptation action across all sectors, this would not only avert the worst health effects, but would also offer additional health gains through clean air, safe water, nutritious food, and safe active travel, thereby protecting lives, health systems, ecosystems, and economies.

In 2025, governments can show their climate and health ambitions with the submission of their latest nationally determined contributions (NDCs)—the third iteration of their climate action plans, which detail their targets for reducing emissions for 2035. At the time of writing, 98 countries, responsible for 72.7% of global emissions, had submitted their new NDCs.^3^ China and the European Union additionally announced a new emission reduction target and target range, respectively, ahead of submitting full NDCs, although these are not aligned with the 1.5°C target.^4–6^ While NDCs are prepared nationally, the International Court of Justice determines that “Parties are obliged to … ensure that their NDCs fulfil their obligations under the Paris Agreement,”^7^ and refers to the interdependence of the human right to a clean, healthy, and sustainable environment with other human rights. Countries therefore need to ensure that health impacts are sufficiently reflected in their latest NDCs. At COP30, governments must jointly decide how to respond to identified gaps in their ambitions to reduce emissions.

Analysis of previous NDCs by the World Health Organization (WHO) found that 91% make some reference to health and 63% have health adaptation actions. But only around 30% identify health benefits of reducing emissions or allocate finance for action at the climate-health intersection.^8^ The Global Climate and Health Alliance’s examinations of previous and recent NDCs indicate that countries that typically produce lower emissions, but endure the most severe health impacts from the climate emergency, dedicate greatest attention to health in their NDCs.^9 10^ The 2024 WHO quality criteria for integrating health into NDCs^11^ provide clear guidance for embedding health in climate planning. But, although some countries acknowledge health in climate planning, most stop short of including measurable targets, financing, or intersectoral coordination mechanisms. We propose that health should be reflected in NDCs by taking the following actions:

- *Align emission reductions with the Paris Agreement, including targets for non-CO_2_ emissions*—To protect and promote health, governments must commit to their “fair share” of emission reductions in line with the Paris Agreement, taking into account historical emissions and present capacities.^12^ Significant disparities in emissions persist, with average per capita emissions in the China, the European Union, Russia, and the United States being higher than the world average, whereas India and Indonesia remain significantly below average.^1^ Notably, almost 50% of the current global temperature rise above pre-industrial times can be attributed to non-CO_2_ greenhouse gases (methane, tropospheric ozone, fluorinated gases, and nitrous oxide) and black carbon. These are often referred to as “super pollutants” on account of their strong heating effect.^13^ Many super pollutants are relatively short lived in the atmosphere, and actions to cut their emissions have a rapid effect on slowing climate change. Reduction in black carbon emissions also yields major health benefits, and methane mitigation reduces the formation of tropospheric ozone also resulting in benefits for health. Reducing nitrous oxide helps protect the ozone layer, which prevents harmful ultraviolet radiation reaching the Earth’s surface as well as improving air quality.^13^
- *Take action in the healthcare sector*—All countries must develop national healthcare adaptation actions, following the example of countries including Bangladesh and Uganda. The healthcare sector contributes about 4-5% of global emissions, particularly from supply chains, and countries, particularly those with high per capita emissions, with necessary capacity should seek to reduce emissions while maintaining and improving quality and accessible care.
- *Optimise health gains across sectors*—To optimise dual wins for people and the planet, NDC mitigation and adaptation actions should be implemented in both the healthcare sector and health determining sectors such as energy, sanitation, agriculture, and transport, taking health as a cross sectoral objective.^14^
- *Ensure support for implementation and monitoring*—Actions to meet the NDCs must be adequately supported by available budgets. This investment would be supported by data quantifying the health impacts of inaction and therefore the costs of doing nothing. The health, economic, and social returns on investment in implementing NDC actions should also be monitored to support the case further.

Achieving these actions will require cross sector coordination between ministries of climate, energy, transport, agriculture, health, housing, finance, and beyond, accompanied by detailed consultations with local and indigenous communities and civil society, engaging them in shaping NDCs and their implementation. Health professionals have a vital role in advocating for stronger integration of health in national climate planning. They must also communicate to decision makers that protecting people and the planet are not separate goals, but deeply interdependent and can bring near term benefits.

The submission of NDCs must be followed by implementation. The United Nations Environment Programme’s *Emissions Gap Report 2025* indicated that nine G20 countries will not achieve their 2030 NDC targets with their existing climate policies.^1^ Several G20 members whose current policies are sufficient to meet their NDC targets have failed to substantially improve their target levels. Several actions can support the implementation of NDC commitments. Firstly, the health benefits of actions to mitigate climate change must be emphasised and made more visible to local and national governments to help motivate action.^14 15^ Well designed policies on carbon pricing aimed at reducing poverty or withdrawal of fossil fuel subsidies that channel resulting government income to support the achievement of health benefits and other socially desirable outcomes, such as universal health coverage or subsidies for renewable energy, could also accelerate the transition to a net zero emission future.^16 17^

Evaluating the impact of each countries’ climate actions on health and climate outcomes is imperative—for example, in the biennial transparency reports submitted to the United Nations Framework Convention on Climate Change—because a dearth of rigorous evaluations results in a lack of accountability and increases risks of “greenwashing” by individual governments.^14^ Adherence to guidance for evaluation of climate actions can improve confidence in findings and accelerate their implementation.^18^ The submission of NDCs is only the beginning. Without clear implementation plans, the targets are worth little. At COP30, governments must ensure that their NDCs are sufficiently ambitious, incorporate health targets, and have clear plans for implementation. In 2026 and beyond, the health community should seek opportunities to engage with governments to maximise the potential health gains of NDC implementation.

Provenance and peer review: Commissioned, not peer reviewed.
